# Chemical Composition, Microstructure, Tensile and Creep Behavior of Ti60 Alloy Fabricated via Electron Beam Directed Energy Deposition

**DOI:** 10.3390/ma15093109

**Published:** 2022-04-25

**Authors:** Guodong Zhang, Wei Liu, Peng Zhang, Huaping Xiong, Jianshi Gao, Huai Yu, Hong Yuan

**Affiliations:** 3D Printing Research & Engineering Technology Center, Beijing Institute of Aeronautical Materials, Beijing 100095, China; zzggdd2010@163.com (G.Z.); liuwei2011621@163.com (W.L.); zpzpup@163.com (P.Z.); gaojianshiuow@163.com (J.G.); yumugui@163.com (H.Y.); hong.yuan@biam.ac.cn (H.Y.)

**Keywords:** near-α titanium alloy, electron beam, directed energy deposition, microstructure, tensile property, creep

## Abstract

Electron beam directed energy deposition (EB-DED) is a promising manufacturing process for the fabrication of large-scale, fully dense and near net shape metallic components. However, limited knowledge is available on the EB-DED process of titanium alloys. In this study, a near-α high-temperature titanium alloy Ti60 (Ti-5.8Al-4Sn-4Zr-0.7Nb-1.5Ta-0.4Si) was fabricated via EB-DED. The chemical composition, microstructure, tensile property (at room temperature and 600 °C), and creep behavior of the fabricated alloy were investigated and compared with those of the conventional wrought lamellar and bimodal counterparts. Results indicated that the average evaporation loss of Al and Sn was 10.28% and 5.01%, respectively. The microstructure of the as-built alloy was characterized by coarse columnar grains, lamellar α, and the precipitated elliptical silicides at the α/β interfaces. In terms of tensile properties, the vertical specimens exhibited lower strength but higher ductility than the horizontal specimens at both room temperature and 600 °C. Furthermore, the tensile creep strain of the EB-DED Ti60 alloy measured at 600 °C and 150 MPa for 100 h under as-built and post-deposition STA conditions was less than 0.15%, which meets the standard requirements for the wrought Ti60 alloy. The creep resistance of the EB-DED Ti60 alloy was superior to that of its wrought bimodal counterpart.

## 1. Introduction

Owing to their high strength-to-weight ratio and the ability to withstand elevated temperatures, titanium alloys are widely used in the aerospace industry, particularly in structural and engine applications [1]. Recently, high-temperature titanium alloys, such as IMI834 [2], Ti-1100 alloy [3] and BT36 alloy [4], have garnered attention in the field of aircraft engines as promising materials for the fabrication of compressor disks and blades undergoing long-term stress at 600 °C. The Ti60 alloy is another new near-α titanium alloy developed in China for service in aero-engine up to 600 °C [5,6]. 

Additive manufacturing (AM) allows structure fabrication in a layer-by-layer deposition process and revolutionizes the manufacturing industry [7,8,9]. Extensive studies have been carried out on the AM process numerical simulation (heat transfer, melt pool and solidification, et al.), microstructure characterization and mechanical properties [10,11,12,13].

The relationship between the microstructure and mechanical properties of Ti60 alloy manufactured using laser-directed energy deposition (L-DED) has been studied [6,14,15]. Chen et al. [14] investigated the relationship between the L-DED laser power input, microstructure, and stress rupture properties of the Ti60 alloy. The formation of a Widmanstätten microstructure was observed when using a high power input, whereas a basket-weave microstructure was observed at a low power input. The Widmanstätten structure exhibited significantly lower stress rupture properties compared to the basket-weave structure, with the former showing distinct intergranular fractures, whereas dimple ruptures were observed in the latter. Zhang et al. [15] compared the microstructure, tensile properties, and stress rupture life of an L-DED-fabricated Ti60 alloy under different post-processing heat treatment conditions. Compared with the as-built alloy, the room temperature tensile ductility of the L-DED-fabricated alloy was improved by 40%, with a 24.3% longer stress rupture life for specimens annealed at T_β_-10 °C. This could be explained by an increase in the β phase content and a discontinuous α colony boundary [15]. Near-equiaxed β grains were successfully generated in an L-DED-fabricated Ti60 alloy with reduced anisotropic tensile strengths without the use of any additives or auxiliary methods [6]. However, so far, electron beam directed energy deposition (EB-DED) of near-α high-temperature (up to 600 °C) titanium alloys (including the IMI834, Ti-1100 and BT36) has not been reported.

EB-DED process offers several advantages, such as high deposition rates of up to 2500 cm^3^/h, approximately 100% feedstock consumption efficiency, and a power-usage efficiency of ~95% [16,17]. It is particularly attractive for the fabrication of large-scale, fully dense and near net shape metallic components [18,19]. However, compared to powder bed fusion and other directed energy deposition processes, EB-DED produces subpar results.

Research on titanium alloys fabricated by AM has been mainly focused on the processing parameters, microstructure, and mechanical properties, such as tensile strength [20], fracture toughness [21], and fatigue [22,23]. Recently, high-temperature creep behavior has gained significant attention as an important property of alloys formed via AM. For example, Kim et al. [24] investigated the effects of heat treatment on the high-temperature compressive creep behavior of the selective laser melting (SLM) fabricated Ti-6Al-4V alloy. Compared to the fully martensite structure of the as-built alloy, the specimens treated at 1040 °C for 1 h followed by furnace cooling (with a Widmanstätten structure) had a low creep strain in all stress ranges (i.e., superior creep resistance). Viespoli et al. [25] reported that the creep performance of the as-built Ti-6Al-4V alloy formed via SLM was equivalent to (or better than) that of its counterparts produced using hot-forging. The creep response of a Ti-6Al-4V alloy produced via SLM was also investigated after annealing at 740 °C for 2 h [26]. Compared to the Ti-6Al-4V alloy produced via conventional technologies, the Ti-6Al-4V alloy formed via SLM exhibited lower creep rates at a given stress at 500 °C in the high-stress regime. For longer-testing durations at temperatures of 500 °C or higher, the material response was equivalent to that of similar alloys produced by conventional technologies. However, the high-temperature mechanical properties of alloys produced via AM have not been extensively researched thus far. In particular, research on high-temperature creep properties remains insufficient despite its significance in high-temperature structural material applications [24]. High-temperature creep is one of the essential primary characteristics of near-α high-temperature titanium alloys as they are intended for use in temperatures up to ~600 °C [27]. To the authors’ best knowledge, there have been no reports thus far on the creep behavior of near-α high-temperature titanium alloy fabricated by AM. Therefore, it is imperative to investigate the high-temperature properties, especially creep, of Ti60 manufactured via AM.

In this study, a near-α high-temperature titanium alloy Ti60 was fabricated using EB-DED. The chemical compositions, microstructures, tensile properties (at room temperature and 600 °C), and high-temperature tensile creep characteristics were investigated in both the vertical (V) and horizontal (H) directions. The results were compared with their conventional wrought lamellar and bimodal counterparts. Furthermore, the influence of chemical composition and microstructure on the mechanical properties was also evaluated.

## 2. Materials and Methods

**Figure 1:**
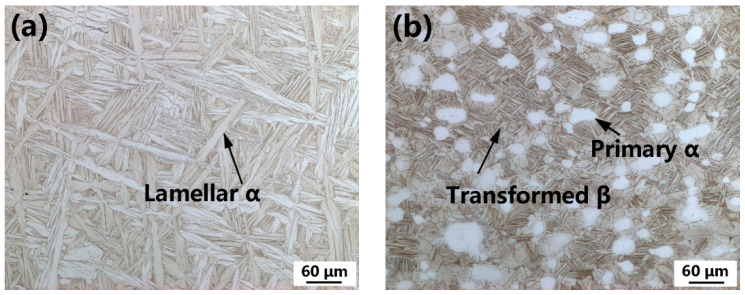
Wrought Ti60 alloy with lamellar (**a**) and bimodal (**b**) microstructure.

The Ti60 alloy with the nominal composition of Ti-5.8Al-4Sn-4Zr-0.7Nb-1.5Ta-0.4Si (wt.%) was received in bar form with a diameter of 300mm, which was manufactured by Western Superconducting Technologies Co., Ltd., Xi’an, China. The β transformation temperature of the wrought bar was metallographically measured to be 1049 °C. The as-received Ti60 alloy bar was subjected to numerous forging passes in the α + β phase field. To obtain the lamellar (Figure 1a) and bimodal (Figure 1b) microstructures, the wrought Ti60 alloy was solution heat-treated at β (1060 °C) and α + β (1030 °C) phase fields, respectively, followed by aging at 750 °C for 2 h. A wire feedstock with a diameter of 1.6 mm was obtained from the wrought bar via forging, rolling, and drawing processes.

A Ti60 alloy block with dimensions of 150 mm (X) × 100 mm (Y) × 80 mm (Z) was fabricated using a KL-106 EB-DED system made by E.O. Paton Electric Welding Institute, Ukraine. The used EBAM system consists of a 2500 mm × 2500 mm × 2500 mm vacuum chamber, movable electron beam gun, wire feeding system, and 5-axis computer numerical control (CNC) table. The wrought Ti60 alloy was used as the deposition substrate. The detailed deposition strategy is described in a previous study [28]. The optimized EB-DED parameters used to obtain a fully dense sample are summarized in Table 1.

The chemical compositions of the wire feedstock and as-built Ti60 alloys were analyzed according to the American Society for Testing and Materials (ASTM) standards. The Al, Sn, Zr, Nb, Ta, Fe, and Si contents in the wire feedstock were analyzed using inductively-coupled plasma atomic emission spectrometry (ICP-AES) and direct current plasma atomic emission spectrometry (DCP-AES) in accordance with ASTM E2371. The C content was determined using the combustion analysis method in accordance with ASTM E 1941. The N and O contents were analyzed using an inert gas fusion (IGF) method following ASTM E 1409. The H content was analyzed using an IGF thermal conductivity/infrared detection method per ASTM E 1447. Twenty specimens were extracted from the as-built samples along the build direction.

The β-transus temperature of the as-built Ti60 alloy was metallographically determined to be approximately 1045 °C, per the BS EN 3684:2007 specification. The round-bar specimens (Φ 13 mm × 73 mm) were subjected to post-deposition solution treatment at 1030 °C for 2 h, followed by air-cooling and aging at 750 °C for 2 h. The solution and aging treatments were conducted in the air in an electrical resistance furnace. The post-deposition heat treatment schedule is consistent with the wrought counterpart [29]. The schematic of the analyzed microstructure and mechanical property specimens is described in the previous study [28].

Metallographic specimens were prepared using standard methods. The microstructures of the specimens formed under as-built and solution treatment with aging (STA) conditions were characterized via optical microscopy (OM; ZEISS Axio Observer 7 optical microscope, Tokyo, Japan) and scanning transmission electron microscopy (STEM) with a high-angular annular dark field (HAADF, FEI Talos F200X G2, Tokyo, Japan) in conjunction with energy dispersive spectroscopy (super X energy dispersive spectrometer system). Image-Pro Plus (version 6.0, Media Cybernetics, Inc., Rockville, MD, USA) was used to measure the thicknesses of the α-phases and precipitates based on pixel counting.

**Figure 2:**
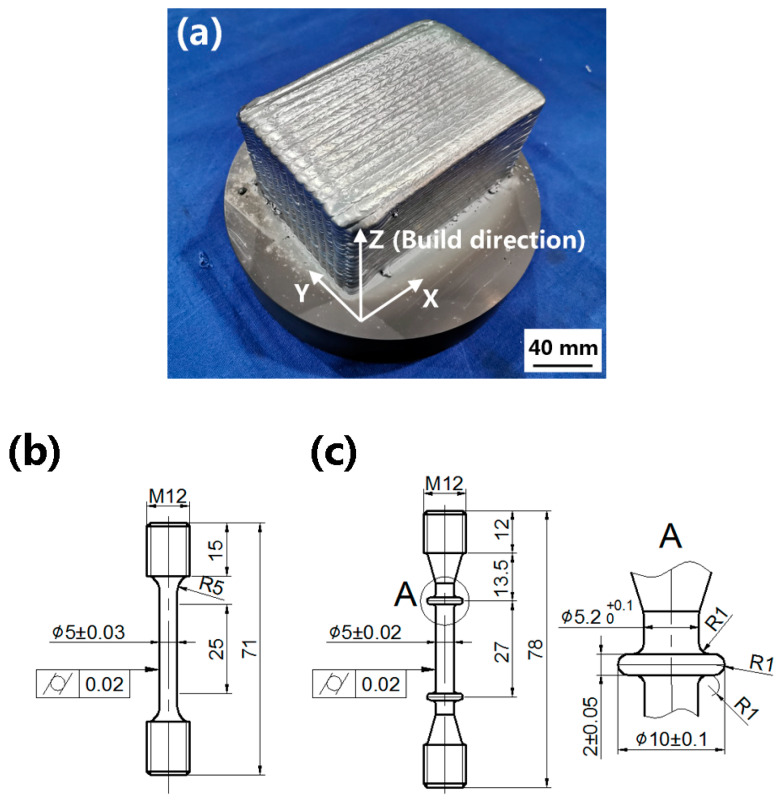
(**a**) EB-DED-formed sample, (**b**) tensile specimen, and (**c**) creep specimen.

Standard cylindrical tensile and creep specimens (Figure 2b,c, respectively) were mechanically machined from the as-built and the STA treated specimens (with surface contamination removed via CNC turning) in both the vertical (V) and horizontal (H) directions. Room-temperature tensile tests were performed according to the ISO 6892-1 specification using an Instron 5887 universal tensile-testing machine. The tensile tests at 600 °C were performed according to the ISO 6892-2 specification using an Instron 5982 machine. The data reported are averaged from triplicate measurements under each condition.

The tensile creep tests were performed at 600 °C with a stress of 150 MPa for 100 h according to the ISO 204 specification using an RWS 100 machine in air. The creep strain was continuously measured for 100 h using a linear variable differential transducer (LVDT) connected to an extensometer mounted to the ridges of the creep sample. During the creep testing, the temperature was controlled to ±0.5 °C in a three-zone furnace using a set of three chronel-alumel thermocouples and proportional–integral–derivative (PID) controllers. Creep tests under each condition were triplicated. STEM was also employed to observe the microstructures of the specimens after creep testing. The samples for the microstructural examination were cut from the plastic deformation area.

## 3. Results and Discussion

### 3.1. Chemical Compositions

**Figure 3:**
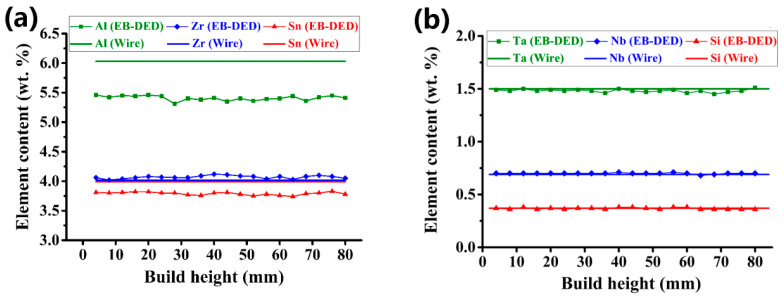
Chemical compositions of the wire feedstock and the as-built alloy along the build direction, Al, Sn, Zr (**a**) and Ta, Nb, Si (**b**).

**Figure 4:**
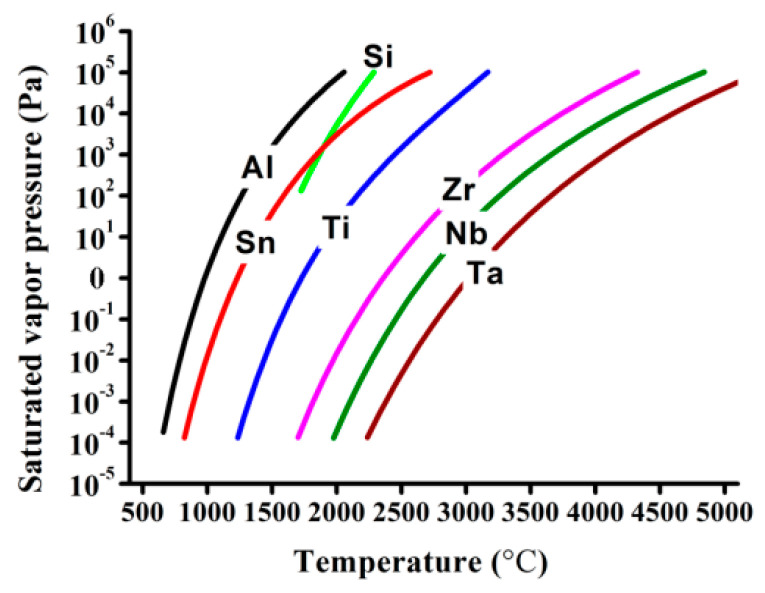
Saturated vapor pressure of pure elements in Ti60 alloy as a function of temperature [39].

Element distribution within the EB-DED Ti60 alloy along the build direction and the elemental composition of the wire feedstock are shown in Figure 3. The selective evaporation of the elements was apparent (Figure 3a), with an average evaporation loss of Al and Sn of 10.28% and 5.01% (Table 2), respectively. The substantial selective evaporation of Al and Sn resulted from (i) the high surface-temperature of the melt pool induced by the high-power density of the electron beam [30,31]; (ii) the vacuum environment [32,33,34]; (iii) the large difference between the respective saturated vapor pressures of different elements [35] (Figure 4). The evaporation loss of Al in the Ti60 alloy was significantly lower than that in Ti-6Al-4V generated using EB-DED by Pixner et al. (~14%) [36] and Xu et al. (up to 39%) [37]. High melting-point elements, such as Ta, Zr, and Nb, were stable. Although Si has a high vapor pressure at elevated temperatures (Figure 4), it did not evaporate because of its low weight percentage in the alloy. Owing to the protective working environment, the N and O contents were consistent with those of the wire feedstock. A significant decrease in the H content (from 0.0044 to 0.0008%; ~82% decrease) was observed. This significant decrease in the H content can be advantageous in preventing pore formation during EB-DED processing. The same line energy (the key parameter affecting vaporization [38]) was used for all of the layers, and a small elemental fluctuation along the build direction was observed (Figure 3).

### 3.2. Macro- and Microstructure Evolution

**Figure 5:**
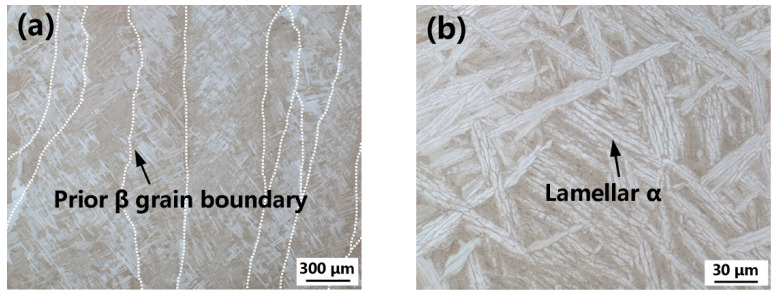
Cross-sectional macrostructure (**a**) and microstructure (**b**) of the as-deposited Ti60 alloy. The prior β grain boundaries in (**a**) were traced in white dots.

The macro and microstructures of the as-built Ti60 alloy are shown in Figure 5. The macrostructure is characterized by coarse columnar grains with an average width of 426 μm. As shown in Figure 5a, columnar grains grew epitaxially through multiple layers. The growth direction of the columnar prior-β grains is inclined ~14° to the build direction (*Z*-axis) and not fully parallel to the deposition direction [28]. In the build direction, the steep thermal gradient of the melt pool provides a perfect selection environment for grain growth. The columnar grains with their preferred grain growth direction aligned with the thermal gradient direction, have a growth advantage, and they are able to kill the adjacent grains during the competitive grain growth process [40]. This type of columnar morphology of prior-β grains has been previously reported in EB-DED Ti-5Al-2Sn-2Zr-4Mo-4Cr [41] and Ti-6Al-2Zr-1Mo-1V alloys [28]. However, compared with the previous study [21,30], a clear variation in the β grain size was observed, which is dependent on the chemical composition and processing parameters, such as power, traveling speed, feed speed, layer thickness, and cooling rate. The epitaxial growth of columnar β grains in the as-built Ti60 alloy can be explained using the thermal gradient generated by the conductive heat flowing downward through the pre-deposited part [42] and the partially melted crystal as the substrate for nucleation to start [40].

**Figure 6:**
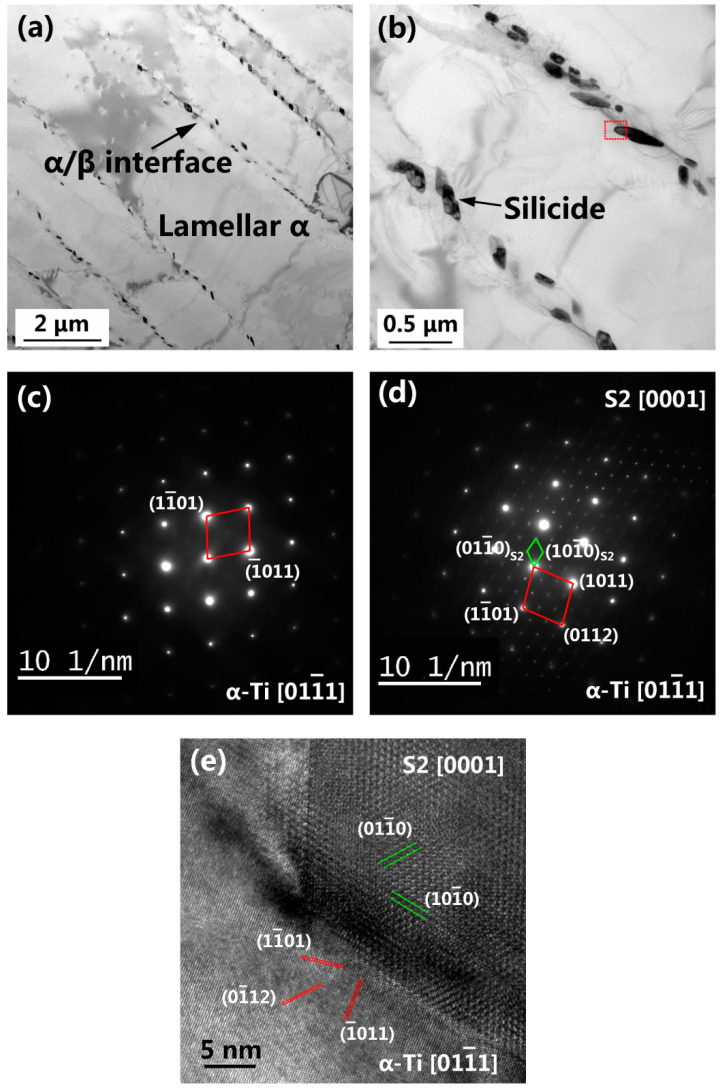
STEM images of the as-built Ti60 alloy: lamellar α (**a**) and silicides (**b**); SADPs of the lamellar α (**c**) and silicides (marked as S2) (**d**), and the HRTEM image of the α-Ti/silicide interface (**e**).

STEM images of the as-built Ti60 alloy are shown in Figure 6. Within the prior β grains, a lamellar α microstructure and numerous elliptical silicides precipitated from the α/β interface (Figure 6a). These silicides preferentially nucleate at the boundary of the α-plates as the β phase (enriched in silicon) is retained between adjacent alpha plates [43,44]. The long axes of the elliptical silicide are nearly parallel to the α/β interfaces, as shown in Figure 6b. The long axis of the silicides had a length of 40~350 nm. No silicide precipitation was observed within the α plates (Figure 6b), suggesting low levels of silicon in the α phase [42]. The average thickness of the lamellar α was 1.19 μm.

**Figure 7:**
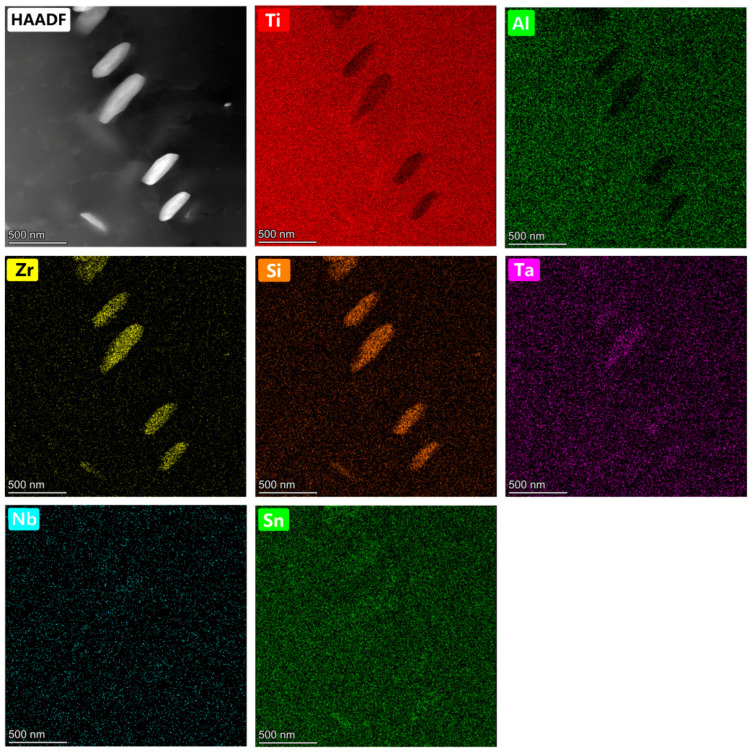
HAADF-STEM image and EDS maps of a typical silicide precipitates region.

Selected-area diffraction patterns (SADPs) of the lamellar α phase (Figure 6c) of the as-built Ti60 alloy exhibited only α reflections, indicating an absence of ordering (α_2_) within the α plates. This observation is inconsistent with the analysis result of the L-DED Ti60 alloy [45]. The SADPs (Figure 6d) and EDS maps (Figure 7) indicated that the precipitates were (Ti, Zr)_6_Si_3_ silicides (S2 type) with the lattice parameters of a = 0.72 nm and c = 0.38 nm, which is congruent with previously reported data for a similar near-α high-temperature titanium alloy [46]. Figure 6e shows a high-resolution TEM (HR-TEM) image derived from the marked zone (red frame) in Figure 6b. The orientation relationship between the silicide and the α-Ti phase was established as (0110)S2//(0112)α-Ti (Figure 6e).

**Figure 8:**
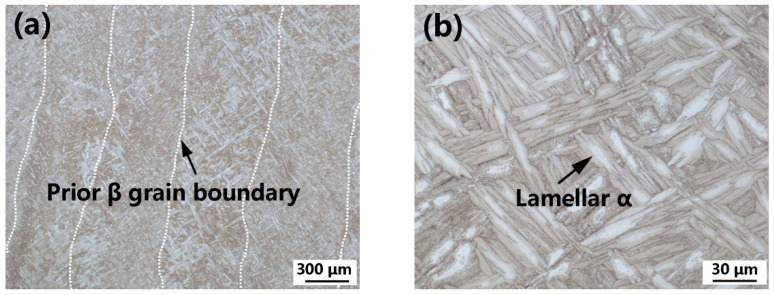
Cross-sectional macrostructure (**a**) and microstructure (**b**) of the post-deposition STA Ti60 alloy. The prior β grain boundaries in (**a**) were traced in white dots.

**Figure 9:**
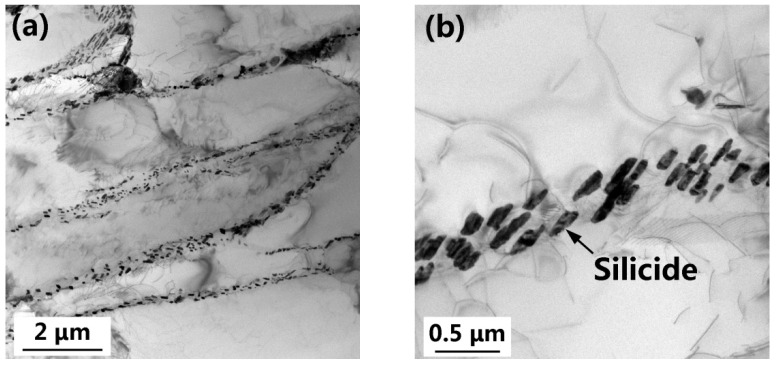
STEM images of the post-deposition STA Ti60 alloy: lamellar α (**a**) and silicides (**b**).

The microstructures of the specimens treated via post-deposition STA are shown in Figure 8. Because of the sub-transus solution temperature, the columnar morphology of the prior-β grains remained unchanged. Compared to the as-built specimen, the lamellar α phase for the STA specimen coarsened significantly (1.19 μm vs. 2.05 μm); this observation is consistent with that of our previous study [28]. In addition, a substantial amount of silicides precipitated at the α/β interface for the specimen treated via post-deposition STA (Figure 9). The size of the silicide precipitates was similar to that of the as-built alloy.

### 3.3. Mechanical Properties

#### 3.3.1. Tensile Properties at Room Temperature and 600 °C

**Figure 10:**
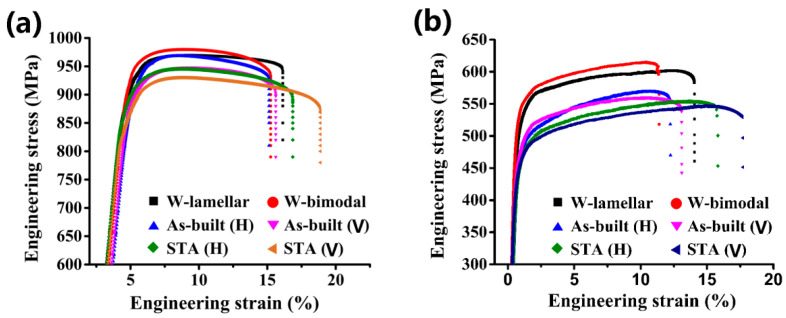
Tensile properties for the EB-DED and wrought Ti60 alloy at room temperature (**a**) and 600 °C (**b**).

Tensile properties for the EB-DED and wrought Ti60 alloys at room temperature and 600 °C are presented in Table 3 and Table 4, respectively. The representative tensile engineering stress-strain curves are presented in Figure 10. 

As expected, the wrought-lamellar (W-lamellar) specimens showed inferior ductility and superior strength compared with the wrought-bimodal (W-bimodal) specimens. Notably, the strength of the specimens produced under as-built and post-deposition STA conditions was lower than that of the wrought alloy. This can be attributed to the larger prior-β grains of the as-built specimens and the post-deposition STA specimens per the Hall-Petch relationship [9] and the significant loss of solid solution-strengthening elements, Al and Sn [47]. 

Compared to the as-built specimens, the post-deposition STA specimens exhibited inferior strength and superior ductility owing to their coarser lamellar α [41]. The H direction specimens exhibited slightly higher strength but lower ductility than the Z direction specimens at room temperature as well as at 600 °C. This anisotropic behavior is also consistent with reported data for titanium alloys fabricated by EB-DED [28], electron beam melting (EBM) [48], SLM [49], L-DED [50], and wire-fed plasma arc directed energy deposition [51]. The anisotropic prior β grain morphology and the presence of the continuous grain boundary α layers are the main causes of the anisotropic tensile properties [52].

**Figure 11:**
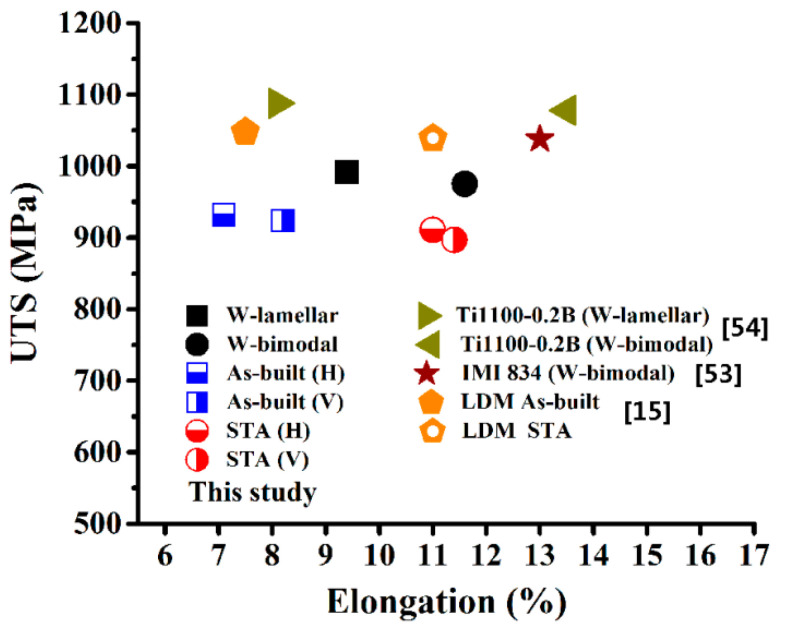
Comparison of the ultimate tensile strength and elongation at room temperature of the wrought and AMed 600 °C high-temperature titanium alloys.

Figure 11 gives a comparison of the ultimate tensile strength and elongation at room temperature of the wrought and AMed 600 °C high-temperature titanium alloys. It can be seen from the comparison that the strength of EB-DED Ti60 alloy is lower than that of the wrought IMI 834 [53], wrought Ti1100 [54], and Laser melting deposition (LDM) Ti-5.54Al-3.38Sn-3.34Zr-0.37Mo-0.46Si alloy [15], but its ductility is comparable to that of the wrought Ti1100 alloy with bimodal microstructure and the LDM Ti-5.54Al-3.38Sn-3.34Zr-0.37Mo-0.46Si alloy. The higher strength of the laser additive manufactured titanium alloy can be related to the martensite α’ phase formed under the high cooling rate in comparison with that of the electron beam additive manufactured titanium alloy [55].

#### 3.3.2. Tensile Creep Properties at 600 °C

**Figure 12:**
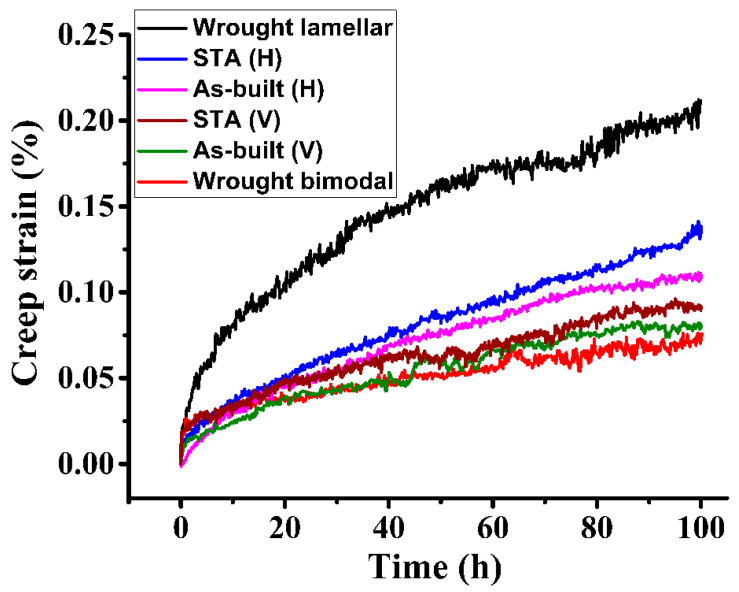
Tensile creep curves for the EB-DED and wrought Ti60 alloys.

The representative creep curves of the EB-DED and wrought Ti60 alloys at 600 °C/150 MPa/100 h are shown in Figure 12, and the creep data are listed in Table 5. The creep curves show the first two stages of the creep process, namely, the primary stage and the steady-state stage. Two distinct behaviors are concurrently observed during the creep process: strain hardening owing to long-range dislocation interaction, and strain recovery because of the thermal activation of short-range dislocation movement. In the primary stage, a gradual increase in the strain recovery with creep deformation is observed, with the creep strain rate gradually decreasing until the steady-state stage is attained [53,56]. 

As expected, the W-bimodal specimen was less creep-resistant compared to the W-lamellar specimen [57]. Similar observations have been previously reported for near-α-Ti alloys [27,56]. The higher creep resistance of the W-lamellar specimen can be attributed to the α/β interfaces acting as barriers for dislocation movements and the larger grains. This reduces the grain boundary sliding and the number of dislocation sources [58]. The partitioning of alloying elements in the lamellar α phase of the bimodal microstructure results in not only lower lamellar matrix strength, compared to the fully lamellar structure, but also a thin layer of the β-phase around the equiaxed α-phase, which provides a high-diffusivity path [27].

The tensile creep strain of the EB-DED Ti60 alloy measured at 600 °C and 150 MPa for 100 h under as-built and post-deposition STA conditions was less than 0.15%, which is comparable to that of its wrought counterpart (≤0.2%). The EB-DED Ti60 alloys (as-built and post-deposition STA) exhibited superior creep resistance compared with the W-bimodal specimen but slightly inferior creep resistance than the W-lamellar. The inferior creep resistance of the as-built and post-deposition STA specimens can be attributed to the loss of solid solution-strengthening elements Al and Sn during EB-DED. This hinders the dislocation motion in the lamellar α phase.

The creep resistance of the post-deposition STA specimens was slightly lower than that of the as-built specimens, which is consistent with the tensile strength results (Table 3 and Table 4). This can be attributed to the substantial precipitation of silicide particles at the lamellar α boundaries during the solution and aging treatments. Si concurrently exists in the solid-solution and precipitate states in titanium alloys [59,60]. However, silicon in the precipitate state has limited activity in pinning dislocations in the case of inhomogeneous distributions. Comparatively, solid solution-state silicon has higher strengthening effects [61]. As shown in Figure 9, a large number of silicide particles is precipitated at the lamellar α boundaries, reducing the Si content in the α matrix and resulting in the deterioration of the creep resistance of the post-deposition STA specimens [62]. The coarsened lamellar α phase in the post-deposition STA specimens also explains the decreased creep resistance [57].

**Figure 13:**
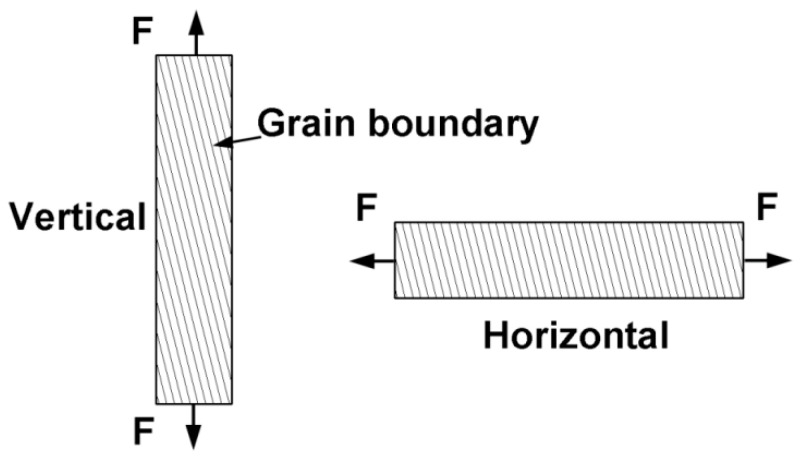
Schematic illustrations of the creep tensile load and grain boundaries for the vertical and horizontal specimens.

Significantly, the creep resistance of the vertical direction specimenswas slightly higher than that of the horizontal direction specimens.This observation is consistent with previouslyreported results from studies investigating SLM-fabricated stainless steel [63] and Ni-base superalloy [64]. The anisotropic creep property can be attributed tothe directional columnar prior-β grains and grain boundary α phase, as illustrated in Figure 13.

**Figure 14:**
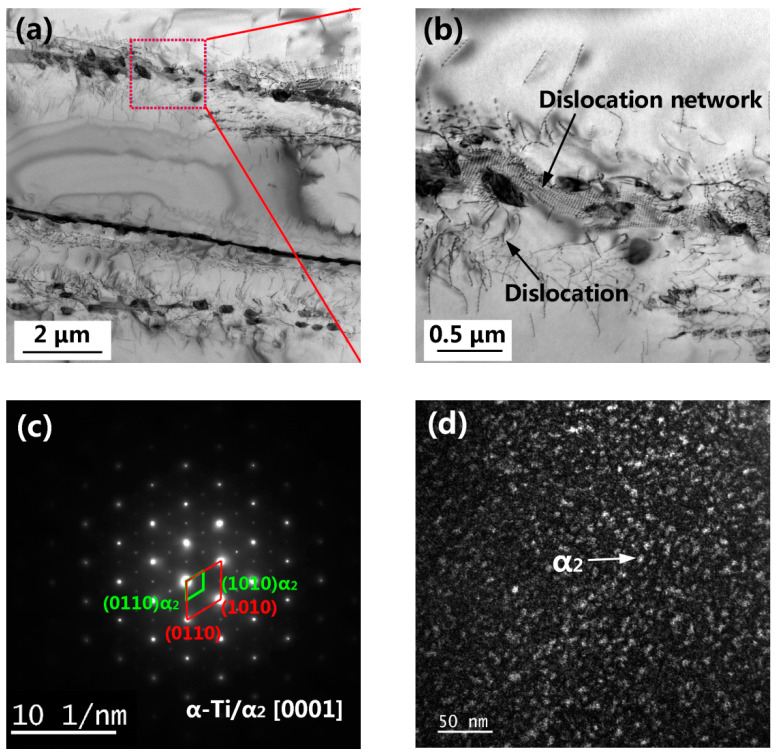
STEM images of the as-built specimen after the creep test (**a**,**b**), SADP obtained from the lamellar α phase (**c**), and dark-field TEM image of the α_2_ phase (**d**).

Figure 14 shows a STEM image of the as-built Ti60 alloy after the creep test. A high density of dislocation segments pinned at the α/β interfaces and silicide particles was observed (Figure 14a,b). During the creep process, the formation of a dislocation network at the α lath boundary was also observed (Figure 14b). SADP obtained from the lamellar α phase of the as-built Ti60 alloy (Figure 14c) shows the presence of weak super-lattice spots, indicating that the ordered α_2_ (Ti_3_Al) phase has precipitated from the α matrix. This is in good accordance with reported data from studies exploring a thermal-exposed laser melting deposited Ti60 alloy [45] and several other high-temperature titanium alloys [46,65]. The dark-field STEM image (Figure 14d) shows the fine and homogeneous dispersion of the α_2_ phase with a diameter of approximately 8 nm.

## 4. Conclusions

In this study, a near-α high-temperature titanium alloy Ti60 (Ti-5.8Al-4Sn-4Zr-0.7Nb-1.5Ta-0.4Si) was fabricated using EB-DED. The chemical compositions, microstructures, tensile properties (at room temperature and 600 °C), and tensile creep characteristics were investigated and compared with those of conventional wrought lamellar and bimodal Ti60 alloys. The influence of chemical composition and microstructure on mechanical properties has been discussed. The main conclusions are as follows:

1. The selective evaporation of elements occurred during EB-DED. The average evaporation losses of Al and Sn were 10.28% and 5.01%, respectively. Compared with the wire feedstock, the H content of the as-built sample significantly decreased from 0.0044 to 0.0008% owing to the high-vacuum environment.
2. The as-built macrostructure was characterized by coarse columnar grains. Within the prior-β grains, a lamellar α microstructure and elliptical (Ti, Zr)_6_Si_3_ silicides precipitated from the α/β interface. The long axis of the silicides had a length of 40~350 nm. The orientation relationship between the silicide and the α-Ti phase was determined as (0110)_S2_//(0112)_α-Ti_. After the post-deposition solution and aging treatment (STA), the α-laths coarsened from 1.19 μm to 2.05 μm. Compared with the as-built specimens, a larger amount of silicides precipitated at the α/β interface of the post-deposition STA specimens.
3. The tensile strength of the as-built and post-deposition STA specimens was lower than that of their wrought counterparts (both lamellar and bimodal microstructures). This is attributed to the larger prior-β grain size and the evaporation loss of elements in the former. Compared to the as-built specimens, the post-deposition STA specimens exhibited inferior strength but superior ductility. The horizontal specimens exhibited slightly higher strength but lower ductility than the vertical specimens at both temperatures (room temperature and 600 °C).
4. The tensile creep strain of the EB-DED Ti60 alloy measured at 600 °C and 150 MPa for 100 h under as-built and post-deposition STA conditions was less than 0.15%, which meets the standard requirements of the wrought counterpart (≤0.2%). The lower Si content in the α matrix and the coarser lamellar α phase in the post-deposition STA specimen explain its inferior creep resistance compared to the as-built specimen. The creep resistance of the EB-DED Ti60 alloy was superior to that of its wrought bimodal counterpart, whereas it was inferior to that of the wrought lamellar specimen.
5. After the creep test, the ordered α_2_ (Ti_3_Al) phases precipitated from the lamellar α matrix, with a high density of dislocations pinned at the α/β interfaces and silicide precipitates.

## Figures and Tables

**Table 1:** EB-DED parameters.

Acceleration Voltage, kV	Beam Current, mA	Travelling Speed, mm/min	Wire Feed Rate, g/min	Hatch Distance, mm	Layer Thickness, mm	Vacuum Pressure, Pa
60	40–50	600–800	30–40	3.5	2.0	4.5 × 10^−2^

**Table 2:** Chemical composition of the wire and EB-DED Ti60 alloy (wt.%).

Materials	Ti	Al	Sn	Zr	Ta	Nb	Si	Fe	C	N	H	O
Wire	Bal.	6.03	3.99	4.02	1.5	0.69	0.37	0.02	0.071	0.003	0.0044	0.073
As-built	Bal.	5.41	3.79	4.07	1.48	0.70	0.37	0.02	0.068	0.003	0.0008	0.073

**Table 3:** Tensile properties for the EB-DED and wrought Ti60 alloy at room temperature.

Materials	Direction	YS/MPa	UTS/MPa	EL/%	RA/%
As-built	H	863	932	7.1	10.3
	V	850	924	8.2	16.8
STA	H	832	911	11.0	20.0
	V	823	897	11.4	22.1
W-lamellar	T	870	992	9.4	17.2
W-bimodal	T	862	975	11.6	19.7

**Table 4:** Tensile properties for the EB-DED and wrought Ti60 alloy at 600 °C.

Materials	Direction	YS/MPa	UTS/MPa	EL/%	RA/%
As-built	H	452	569	11.4	25.9
	V	441	558	12.7	32.2
STA	H	433	554	15.1	30.9
	V	422	543	16.0	36.1
W-lamellar	T	516	617	11.6	26.9
W-bimodal	T	502	602	13.5	39.6

**Table 5:** Creep strain and steady state creep rate of the EB-DED and wrought Ti60 alloys.

Condition	Direction	Initial Strain (%)	Total Strain (%)	Creep Strain (%)	Steady State Creep Rate (s^−1^)
As-built	H	0.159	0.298	0.139	1.63 × 10^−7^
	V	0.169	0.252	0.086	1.32 × 10^−7^
STA	H	0.242	0.391	0.149	2.05 × 10^−7^
	V	0.138	0.235	0.097	1.68 × 10^−7^
W-bimodal	T	0.131	0.356	0.225	2.41 × 10^−7^
W-lamellar	T	0.166	0.240	0.074	1.09 × 10^−7^

## Data Availability

Not applicable.
